# Non-expert listeners show decreased heart rate and increased blood pressure (fear bradycardia) in response to atonal music

**DOI:** 10.3389/fpsyg.2015.01646

**Published:** 2015-10-28

**Authors:** Alice M. Proverbio, Luigi Manfrin, Laura A. Arcari, Francesco De Benedetto, Martina Gazzola, Matteo Guardamagna, Valentina Lozano Nasi, Alberto Zani

**Affiliations:** ^1^Department of Psychology, University of Milano-BicoccaMilan, Italy; ^2^Conservatory of Music “Lucio Campiani”Mantova, Italy; ^3^Institute of Molecular Bioimaging and Physiology – National Research CouncilMilan, Italy

**Keywords:** neuroesthetics, music perception, heart rate, dissonance, auditory processing, emotions, empirical musicology, psychophysiology

## Abstract

Previous studies suggested that listening to different types of music may modulate differently psychological mood and physiological responses associated with the induced emotions. In this study the effect of listening to instrumental classical vs. atonal contemporary music was examined in a group of 50 non-expert listeners. The subjects’ heart rate and diastolic and systolic blood pressure values were measured while they listened to music of different style and emotional typologies. Pieces were selected by asking a group of composers and conservatory professors to suggest a list of the most emotional music pieces (from Renaissance to present time). A total of 214 suggestions from 20 respondents were received. Then it was asked them to identify which pieces best induced in the listener feelings of agitation, joy or pathos and the number of suggested pieces per style was computed. Atonal pieces were more frequently indicated as agitating, and tonal pieces as joyful. The presence/absence of tonality in a musical piece did not affect the affective dimension of pathos (being touching). Among the most frequently cited six pieces were selected that were comparable for structure and style, to represent each emotion and style. They were equally evaluated as unfamiliar by an independent group of 10 students of the same cohort) and were then used as stimuli for the experimental session in which autonomic parameters were recorded. Overall, listening to atonal music (independent of the pieces’ emotional characteristics) was associated with a reduced heart rate (fear bradycardia) and increased blood pressure (both diastolic and systolic), possibly reflecting an increase in alertness and attention, psychological tension, and anxiety. This evidence fits with the results of the esthetical assessment showing how, overall, atonal music is perceived as more agitating and less joyful than tonal one.

## Introduction

The aim of the study was to investigate the possible effects of atonality music and emotional characterization on autonomic responses. While many studies have explored the neural mechanisms of music perception by measuring electrophysiological, neuroimaging, or physiological parameters during music listening (Koelsch, 2014), so far little attention has been paid to the effect of musical style, particularly the presence or absence of tonality, on the esthetic experience. For example, some studies defined contemporary atonal music as “adiabatic”; that is, incapable of transmitting emotions (Frova, 2008). Other scholars have defined it as “entropic” (random, disordered, information-less; see Donin and Feneyrou, 2013), but few studies have been carried out in the field of neuroscience.

Generally speaking, from a perceptual point of view two main differences exist between tonal and atonal music. (1) The presence of a clear and predictive harmonic structure in the former, and (2) the presence of acoustic dissonances in the latter. Overall, tonal pieces are perceived as more congruent and less unexpected than atonal pieces, which violate consonance rules and general music expectations about structure and harmony (e.g., Meyer, 1956; Pearce and Wiggins, 2012). Indeed, it is more difficult to identify the musical structure of atonal pieces, especially for non-expert listeners (Daynes, 2011), and this may reduce music appreciation. On the other hand, experienced listeners attending to serial music may indeed be able to detect certain aspects of its artificial compositional grammar (e.g., the eschewal of pitch repetition), or other types of structures (Ockelford and Sergeant, 2012) not necessarily designed by the composer. This greater understanding is associated with a greater esthetic appreciation. Violations and confirmations of musically induced expectations may be able to affect related psychophysiological activations. In an interesting study Egermann et al. (2013) found a general increase of physiological arousal to unexpected musical events or endings (e.g., note’s pitch) in terms of heart rate and facial expressivity, thus showing how music structure *per se* can heavily affect induced feelings, regardless of music emotional characterization.

Tonal and atonal music also differ considerably from the perceptual point of view, because the latter is full of dissonances and inharmonic chords. It was shown that the perception of inharmonic isolated sounds or chords elicit physiologically different responses in newborns (Perani et al., 2010; Virtala et al., 2012) and human adults (Bidelman and Krishnan, 2009; Bidelman and Heinz, 2011), as well as in other species, such as macaques (*Macaca fascicularis*, Fishman et al., 2001), chimpanzees (*Pan troglodytes*, Sugimoto et al., 2010) and rats (*Rattus norvegicus*, Crespo-Bojorque and Toro, 2015). Although there is some evidence that listening to tonal or atonal music partially activates different brain regions (Minati et al., 2009), the neurophysiological correlates of perceiving atonal music remain a rather unexplored matter.

Zentner et al. (2008) analyzed participants’ reports of felt emotions in response to music listening. The factorial analysis revealed nine esthetic dimensions: wonder, transcendence, nostalgia, tenderness, peacefulness, joyful activation, tension, sadness, and power. It is very likely that many felt emotions shared some of their psychophysiological and neurophysiological substrate. And indeed, Vuilleumier and Trost (2015) showed that complex music-induced feelings (such as wonder, nostalgia, or tenderness) shared common neural substrates. Feelings of power (i.e., being heroic and triumphant) were correlated with the activation of the ventral striatum, similar to the effect of joy but with distinctive increases in the motor cortex and dorsal basal ganglia. On the other hand, wonder (i.e., feeling allured and amazed) also activated the ventral striatum, but unlike joy and power, it activated the motor areas to a lesser degree and the right hippocampus to a greater degree. Nostalgia and tenderness were found to activate the hippocampus, and nostalgia produced increased activity in the brain regions involved in visual imagery. Overall, the available literature suggests that the stimulation of feelings by music might solicit complex but shared associations based on partially overlapping neural mechanisms (Peretz and Zatorre, 2003; Brattico and Pearce, 2013). On the other hand, joy, fear and sadness, among the emotions more commonly studied in research on music and emotion (Zhao and Chen, 2009; Huron, 2013; Kawakami et al., 2013; Liégeois-Chauvel et al., 2014; Hopyan et al., 2015) are pretty distinctive in terms of their biological substrates.

In this study, to possibly detect some changes in autonomic parameters during music listening, only music-induced affective categories that were quite distinctive in terms of their biological substrates were considered, namely: joy, pathos, and agitation. These categories are based on a rough subdivision in: (i) positive mood (joy, well-being, good mood, satisfaction, pleasure), associated with a cholinergic autonomic response, and, neurally with the activation of the ventral tegmental area (VTA) and striate cortex, the dopaminergic reward circuitry, the orbitofrontal cortex, nucleus accumbens (Blood and Zatorre, 2001; Koelsch et al., 2006); (ii) pathos (sadness, pain, commotion, nostalgia, tenderness, sympathy), associated with the activation of the insula, cingulate cortex, ventromedial prefrontal cortex, and hippocampus, along with high prolactin concentrations (Janata, 2009; Trost et al., 2012; Huron, 2013); and (iii) agitation (surprise, fear, tension, excitement, anxiety), associated with an adrenergic sympathetic autonomic response, and the activation of the amygdala, motor cortex, cerebellum, etc. (Koelsch et al., 2006; Gosselin et al., 2007; Brattico and Pearce, 2013; Stupacher et al., 2013).

As for the effects of music-induced feelings on the autonomic nervous system (ANS) the available psychophysiology literature is rather inconsistent (Krabs et al., 2015). For example, some studies demonstrated an increase in heart rate with arousing music and a decrease with tranquilizing music (e.g., Bernardi et al., 2006), and others reported increases in heart rate with arousing as well as tranquilizing music (e.g., Iwanaga et al., 2005). Thus, one intervening factor might be the music-evoked emotional valence. Several studies reported that compared to negative valence (displeasure), positive valence (pleasure) is associated with higher heart rate (e.g., Sammler et al., 2007). However, other studies (e.g., Etzel et al., 2006) failed to find effects of music-evoked emotional valence on heart rate. Again, it has been shown that listening to music can reduce pain intensity and systolic blood pressure in patients during post-operative recovery (Chen et al., 2015). Furthermore, it can reduce stress levels and heart rate in patients with coronary heart disease and cancer (Tan et al., 2015), but no reductions in heart rate or blood pressure in healthy controls were caused by listening to music. In another study by Radstaak et al. (2014), it was shown that although listening to both relaxing and happy music improved subjects’ moods, it did not diminish systolic blood pressure. In another psychophysiological study by Tan et al. (2014), the effect of relaxing music on the recovery of heart rate after exercise was investigated. Twenty-three healthy young volunteers exercised on a treadmill and were then assessed for heart rate recovery and subjected to saliva analysis. The participants were either exposed to sedating music or to silence during the recovery period immediately following the treadmill exercise. No differences were found between exposure to music or silence with respect to heart rate recovery, resting pulse rate, or salivary cortisol, but again, no effect of tonal music was explored. It should be considered that in all studies taken into account the music listened to was relaxing, sedating or tranquilizing in nature, therefore one might expect to find cholinergic responses associated with listening to this sort of music. On the other hand, a parasympathetic defensive reaction such as the so called fear-induced “bradycardia” (Hermans et al., 2013) has not been reported, so far, as a result of listening to classical music. It is commonly produced by the abrupt presentation of intense, noxious or scary sounds, both in animals (Young and Leaton, 1994; Kalin et al., 1996) and in humans (e.g., Anderssen et al., 1993). A fear bradycardia has also been observed during perception of scary or violent movie scenes (Palomba et al., 2000).

The effect of atonality on autonomic parameters has been rarely investigated, except for a study in which the respiratory sinus arrhythmia was measured in 40 mothers and their infants while listening to tonal and atonal music (Van Puyvelde et al., 2014). The authors found no effect of atonal music in mothers but only in infants. Again, Krabs et al. (2015) compared listening to pleasant tonal and unpleasant dissonant music with silence, and found an increase in heart rate during music listening vs. silence but no effect of music pleasantness (and dissonance) on the cardiac response.

In our study, to select tonal or atonal pieces characterized by a given emotional connotation, a group of musicians was asked to indicate some representative pieces that best expressed a given emotion: the number of suggested pieces per emotion and musical style was calculated. The professional suggestions that overlapped more coherently were selected for the experiment. The physiological responses of large group of non-musicians with no education and little interest in music were measured while they listened to music excerpts of various styles (specifically comparing tonal vs. atonal music). In fact, one of the main problems with this type of investigation is that esthetic preferences are strongly mediated by music exposure and education. We assumed that because our participants were not educated and not particularly interested in classical music, they would have been casually exposed to tonal and atonal music through random listening in public places, where music is often played to create an atmosphere or reduce stress. Indeed, in everyday life, we happen to involuntarily (and forcedly) listen to many different types of background music (from pop to jazz, techno to classical) while watching television commercials, waiting on hold on the phone, or when we enter a bar, a restaurant, an airport, or a mall. Many television programs and movies are associated with an atonal soundtrack (especially dramas, horror movies, and thrillers, or when shocking revelations are to be expressed). Therefore, in this study, the effect of “exposure to music” was not completely annulled because participants (university students living in the Milan metropolitan area) belonged to a specific Western society. However, it should be known that the study’s aim was not to establish to what extent the esthetic appreciation depended on evolutionary, anatomical or physiological constraints or whether it was influenced by cultural, historical, and individual differences (Konecni, 1979; Jacobsen, 2010). To follow an ecological approach, we chose to make the participants listen to real tonal or atonal artworks and masterpieces, instead of presenting them simple melodies, chords, or fragments of little artistic value (see Van Puyvelde et al., 2014; Krabs et al., 2015).

## Esthetic Evaluation

### Judges

A paper questionnaire was given to teachers in the local conservatory of music and an email questionnaire was sent to about 50 music professionals whose email addresses were found in the homepages of CIDIM (database of Italian musicians) and Italian music conservatories. Twenty musicians responded enthusiastically and were recruited for the study. They were 20 professional conductors, composers and professors of various Italian conservatories whose mean age varied between 50 and 60 years. The prestigious and excellent careers of all judges included intensive teaching, national and international performances in famous theaters, CD recordings, live television recordings, masterclasses, and award.

### Stimuli and Procedure

The judges were asked to freely provide a list (between 5 and 15) of the most emotional classic instrumental music pieces from the tonal and atonal repertoire. Instructions were: “Please kindly indicate in the relative boxes the musical pieces that, based on your personal experience, are emblematic of three affective categories: agitating, happy, or touching music.”

*Agitating* was defined as music that transmitted anxiety, distress, fear, agitation, and tension.

*Happy* was defined as music that transmitted a good mood, wellness, joy, and happiness.

*Touching* was defined as music that transmitted pathos, grief, melancholy, pain, sadness, nostalgia, and sympathy.

*Tonal* music was defined as any musical production that had a tonal center around which the melody and harmony were based, including the monodic productions of the Middle Ages.

*Atonal* music was defined as any musical production (roughly dated after 1910: from Schönberg onward) that avoided a tonal center or used multiple tonal centers simultaneously. After their initial selection, movie soundtracks, opera pieces and excessively popular pieces were discarded.

A total of 207 suggestion was received: 147 tonal pieces and 60 atonal pieces. Similarly to Kallinen (2005), who asked composers and Conservatory teachers to nominate musical works that expressed specific emotions, we found that a higher proportion of tonal works were selected than non-tonal works. As expected, the music professionals suggested more music based on the diatonic major/minor than the amodal/atonal or dodecaphonic system (i.e., Renaissance, 21th-century).The corpus of suggestions was re-submitted to judges, asking them to identify the most touching, joyful, or agitating pieces among the ones listed (if any, and according to their esthetic preference). Unlike in Kallinen (2005) study, in which suggestions for typical basic emotional music excerpts overlapped minimally (i.e., there were only a few pieces that were mentioned more than once or twice), many pieces received a coherent categorization from the judges, in our study.

### Data Analysis

**FIGURE 1:**
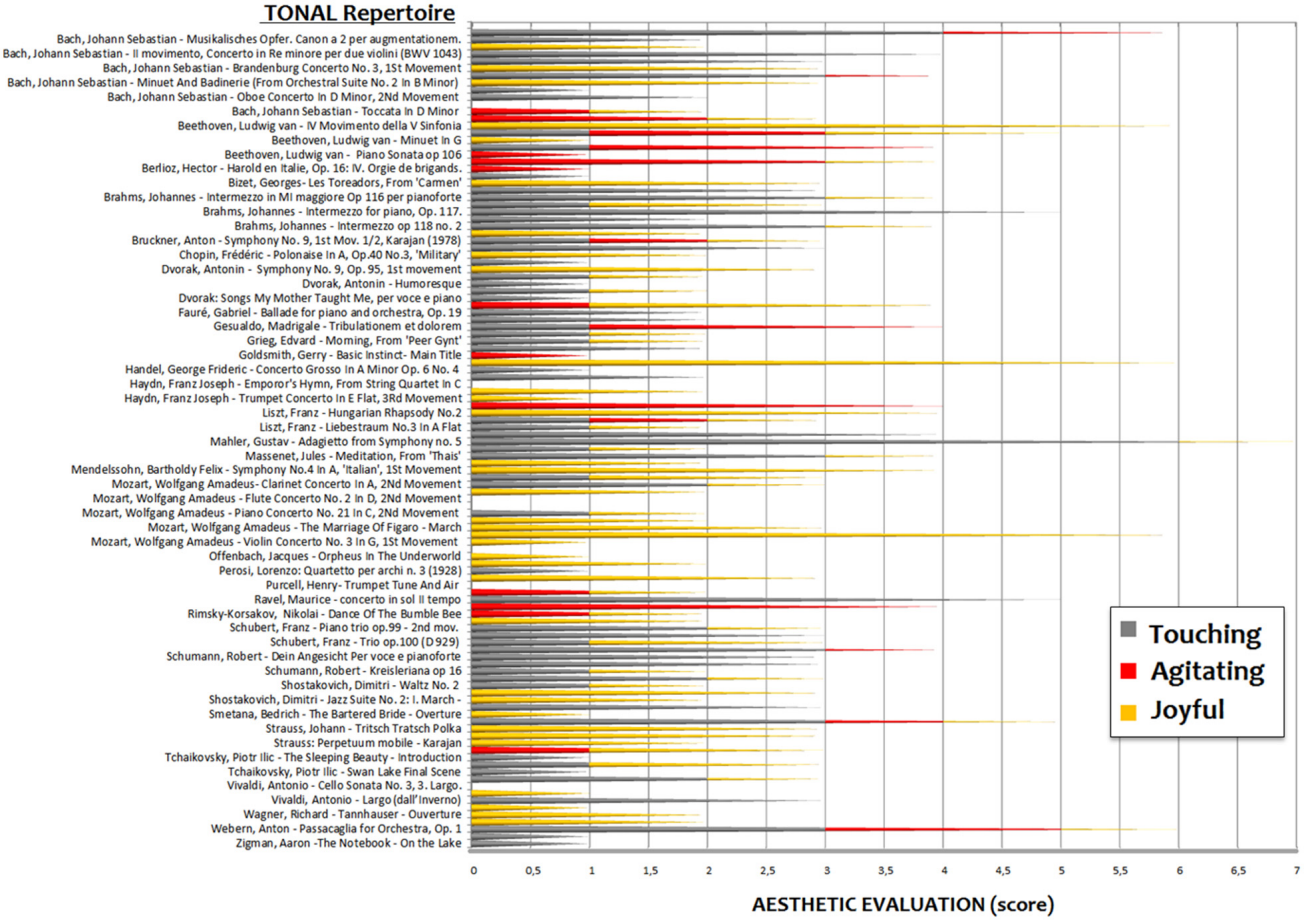
**Esthetic assessment of tonal artworks with respect to the three emotional categories (according to the evaluations of 20 conductors, composers, and conservatory professors).** As in Kallinen (2005) emotional categorization showed a marked inter-individual variability. However, (unlike in the above study, in which there were only a few pieces that were mentioned more than once or twice to illustrate a given emotion), many pieces were categorized in an extremely coherent manner.

**FIGURE 2:**
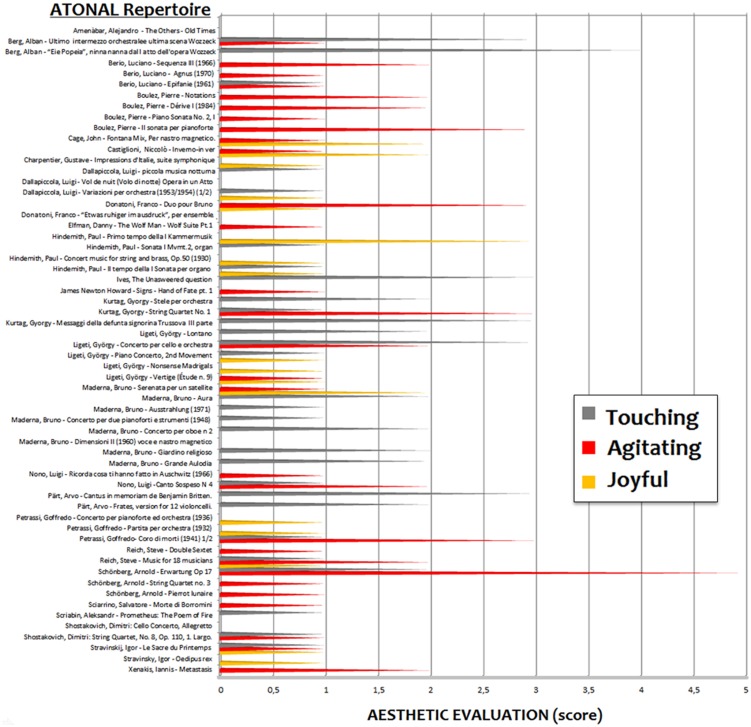
**Esthetic assessment of atonal artworks with respect to the three emotional categories (according to the evaluations of 20 conductors, composers, and conservatory professors)**.

Overall, the 20 judges provided 123 esthetic evaluations (about six pieces per judge) for the 60 atonal pieces and 319 evaluations (about 16 pieces per judge) for the 147 tonal pieces (**Figures 1** and **2**). The number of suggested pieces per emotion and musical style was calculated and descriptive statistical methods and Wilcoxon testing were used to analyze the data. The evaluations were attributed as follows: 12.2% of pieces were attributed to agitating, 42% to touching and 45.8% to joyful categories for the tonal repertoire; 39% of pieces were attributed to agitating, 43.1% to touching and 17.89% to joyful categories for the atonal repertoire.

**FIGURE 3:**
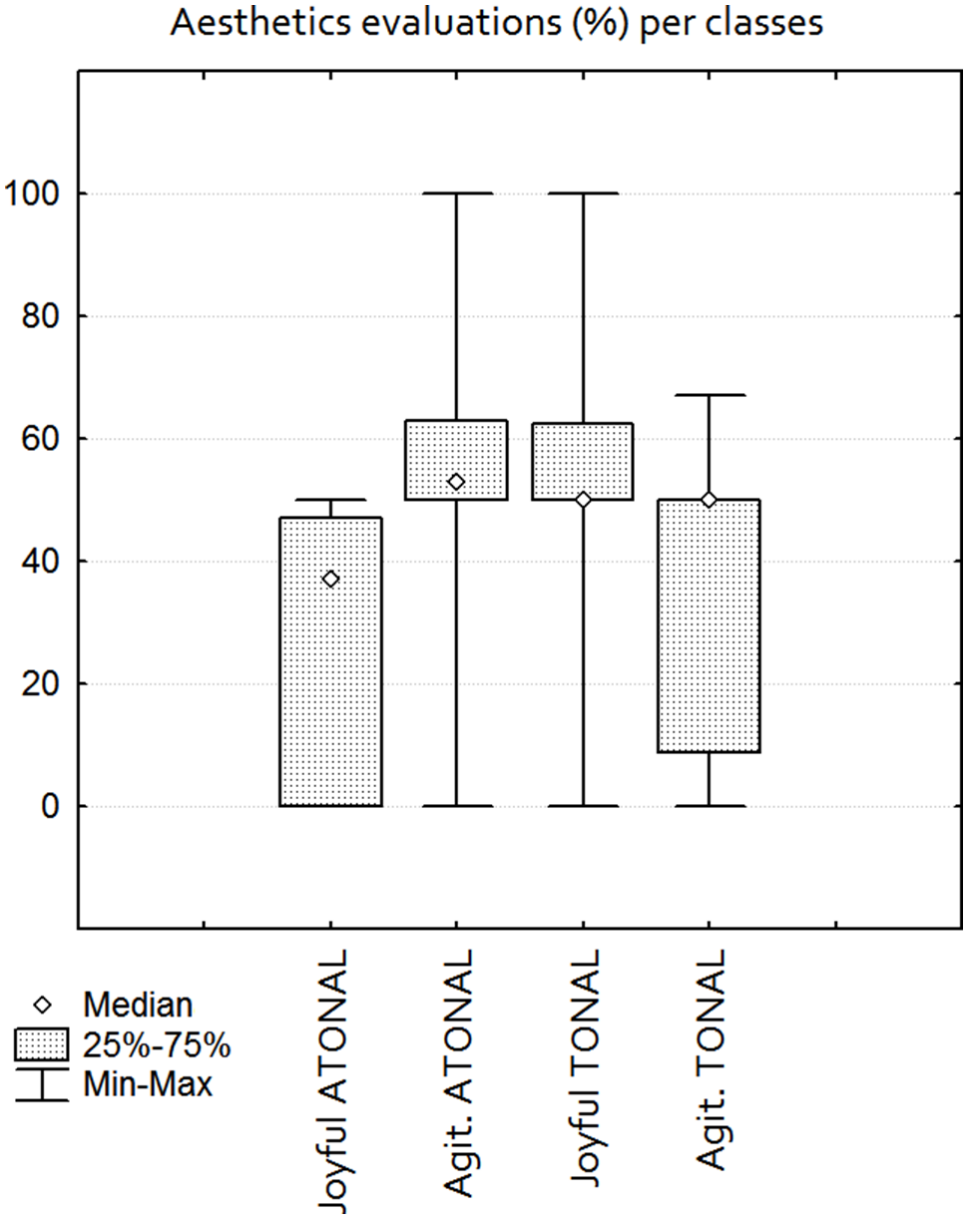
**Results of the paired-samples Wilcoxon rank-sum test applied to the percentage of agitating vs. joyful evaluations attributed to pieces belonging to the two repertoires.** Atonal pieces were judged to be significantly more *agitating* (i.e., as inducing tension) than tonal pieces, which were more frequently labeled as *joyful*. No difference whatsoever was found in the frequency with which a piece was judged to be *touching* (i.e., able to induce pathos) across the two repertoires. This finding is in contrast with the conclusions advanced by Kallinen (2005) that: (i) modern music does not intend to portray emotions; (ii) the meaning of musical emotional qualities in modern music is not as well-established as it is with regard to older music.

As can be clearly appreciated, although the evaluation of pieces as “touching” was not affected by the tonal/atonal dimension (42 vs. 43.1%), scores were asymmetrically distributed to the other emotional categories for the two repertoires. Atonal pieces were judged, overall, to be significantly (*z* = 2.37, *p* < 0.018) more distressing (agitating) than tonal pieces, as shown by a paired-samples Wilcoxon rank sum test applied to the percentage of evaluations attributed to the agitating vs. joyful classes. For tonal pieces the pattern looked the opposite (please see **Figure 3**) but the difference did not reach statistical significance (*z* = 1.48, *p* = 0.13).

### Exclusion/Inclusion Criteria

The most coherently evaluated pieces were examined for their similarities in structure, rhythm, and ensemble instrumentation across the two classes of tonal and atonal repertoires. **Table 1** reports the pieces that received the largest number of coherent evaluations. The selected pieces are highlighted in bold. Based on the constraints imposed by the experimental paradigm, a single piece was selected for each stylistic and emotional category according to a set of criteria that guaranteed intracategoric homogeneity. These were:

**Table 1:** List of musical pieces selected more frequently and coherently by judges to represent each stylistic and affective category.

Composer	Title	Year	Score
**Atonal joyful**			
**Hindemith, Paul**	**First Movement of I Kammermusik** (the fragment was taken from the opening passage)	**1922**	**3**
Cage, John	Fontana Mix, for magnetic tape	1958	2
Maderna, Bruno	Serenade for a satellite	1969	2
Castiglioni, Niccolò	Inverno in-ver	1973	2
**Atonal touching**			
Berg, Alban	Eia Popeia, Lullaby from the first act of Wozzeck	1922	4
**Pärt, Arvo**	**Cantus in Memoriam of Benjamin Britten** (the opening passage, after the first 15 s)	**1977**	**3**
Ives, Charles	The Unanswered question	1906	3
Berg, Alban	Last orchestra interlude from the last scene of Wozzeck	1922	3
Ligeti, György	Concert for cello and orchestra	1966	3
Kurtag, Gyorgy	Messages of the Late Miss R.V. Troussova, Op. 17, 3rd part	1980	3
**Atonal agitating**			
Schönberg, Arnold	Erwartung, Op. 17	1909	5
**Donatoni, Franco**	**Duo Pour Bruno** (ninth panel, from 14′ 37″)	**1975**	**3**
Petrassi, Goffredo	(Chorus of the Dead) Coro di morti	1941	3
Boulez, Pierre	Second piano sonata	1948	3
Kurtag, Gyorgy	String Quartet No. 1	1959	3
**Tonal joyful**			
**Beethoven, Ludwig**	**Fourth Movement of Symphony No. 5 in C major (op. 67)** (last minute of The coda: Allegro)	**1808**	**6**
Haendel, George Friedric	Messiah HWV 56, part 2: Hallelujah in D major	1742	6
Mozart, Wolfgang Amadeus	The Marriage of Figaro – Overture in D major (K492)	1786	6
Mendelssohn, Bartholdy Felix	First Movement of Symphony No.4 “Italian” in A major (op.90)	1834	4
Liszt, Franz	Hungarian Rhapsody No.2, S.244/2 in C-sharp major	1847	4
**Tonal touching**			
Mahler, Gustav	Symphony no. 5 in F major (Adagietto)	1902	6
Brahms, Johannes	Intermezzo for piano in B flat minor (Op. 117)	1892	5
Ravel, Maurice	Second movement of concert in G major	1931	5
**Bach, Johann Sebastian**	**Second Movement of Concert for two Violins in D minor (BWV 1043)** (from measure 10)	**1723**	**4**
Bach, Johann Sebastian	First part of Matthäus Passion in E minor (BWV 244)	1727	4
Mahler, Gustav	Der Abschied from “The Song of the Earth” in C-minor	1909	4
**Tonal agitating**			
Holst, Gustave	The Planets: Mars, the bringer of war in C minor (op. 32)	1916	4
Gesualdo da Venosa, Carlo	Tribulationem et dolorem. Sacrarum cantionum liber primus, in A minor (S1.4)	1603	3
**Bach, Johann Sebastian**	**ST. John Passion in G minor (BWV 245)** (the opening passage)	1724	3
Beethoven, Ludwig van	Piano sonata No. 14 in C-sharp minor, First Movement. (op.27, No.2)	1801	3
Beethoven, Ludwig van	Symphony No. 5, First Movement in C minor (op. 67)	1808	3

*Pieces with the same score are chronologically ordered. In bold are the selected pieces, and in parentheses is indicated which 1 min excerpt was taken.*

(a) Absence of the human voice. On the basis of this principle, “Eia Popeia” by Alban Berg (from the atonal-touching category) was discarded. “Erwartung” by Arnold Schönberg (from the atonal-agitating category) was also discarded.

(b) Composition and size of the instrumental ensemble. On the basis of this principle, piano solo pieces were discarded (Brahms’s intermezzo for piano, Ravel’s concert in G major) from the tonal-sad category.

(c) Tempo and rhythmic structure. On the basis of this principle, Bach’s (and not Mahler’s) and Parvo’s (and not Ives’s) touching pieces were selected as more comparable to each other. Please see here the technical ratio^1^.

(d) Stylistic distinctiveness. According to this criterion, “The Planets: Mars, the Bringer of War in C minor” was discarded in that it is not completely tonal in nature. This piece seems to have an undefined tonal structure. It ends with the C chord using only the root and the fifth, and without the third. Therefore, the chord could be interpreted to be either C-major or C-minor. Holst uses only triads, which follow none of the traditional harmonic progressions according to the diatonic scale degree. Db-major appears to be the tonal center together with the basso ostinato on the note G. Both the Db-major and the basso ostinato on the note G appear to promote the idea of bitonality (Leelasiri, 2001).

### Stimuli and Material

For each of the selected pieces, taken from commercially available CDs, 1 min of connotative track was obtained by cutting the soundtrack via *mp3 DirectCut* and *Format Factory* software. Audio clips were faded at the end (in the last second) via *Audacity* software. Sound tracks were accurately matched for intensity with *MP3Gain* software (89.0 dB).

The six selected pieces were evaluated for their familiarity by a group of 10 university students (mean age = 22.7 years) from the same cohort of experimental subjects (i.e., non-musicians with no musical training) using a three-point scale (0 = no familiarity; 1 = somewhat familiar; 2 = familiar). Instructions informed subjects not to rate the piece by its typology (e.g., classical music, contemporary music, Baroque music) but specifically to rate the piece’s familiarity. In pieces that had some familiarity, subjects were asked to describe the exact circumstances in which they originally heard the piece (e.g., at the mall, at the airport, on TV, during a party, a concert, listening to a CD, MP3, etc.). The scores were submitted to ANOVA whose factors were style (tonal vs. atonal) and emotional tone (sad, happy, agitating). The musical pieces turned out to be very unfamiliar with no differences across classes (mean score = 0.3).

## Psychophysiological Study

### Subjects

Fifty healthy participants (25 males and 25 females), ranging in age between 18 and 28 years (mean age = 22.2 years), took part to the study. They were all right-handed with normal hearing and vision and none had suffered from previous or current psychiatric or neurological diseases. Participants were recruited through *Sona System* (a system for recruiting students who earn credit for their psychology courses by participating in research studies), received academic credits for their participation and provided written informed consent. The experiment was performed in accordance with the relevant guidelines and regulations and was approved by the Ethical Committee of the University of Milano-Bicocca. The participants were blinded to the purpose of the experiment. None of the participants was a musician, and none of them had ever studied music, played a musical instrument, or had a musical activity as a hobby or specific interest. This information was specifically ascertained through the administration of a detailed questionnaire.

### Procedure

Subjects were comfortably seated in an anechoic chamber in dimly lit conditions. They wore headphones and a wrist device (on the left hand) for measuring heartbeat, and blood pressure (Diastolic = DiaBDLP and systolic = SysBDP) whose properties were: INNOFIT(INN-001) model, MWI technology (Precision: BLP ± 3 mmHg, heart rate ± 5%; range: 0–299 mmHg, 40/180 bpm). Participants faced a PC screen (through a mirror) located outside the cubicle, where they observed 300 faces to be remembered in a subsequent recall study. This kept their alertness levels even across the study. Faces were balanced for affective valence and perceived arousal across auditory blocks. The auditory stimulus was delivered through MP3 players. Each musical fragment was heard three times by each participant at different stages of the recording session. To limit the length of the experiment, half of the subjects were randomly assigned to the tonal or atonal conditions. The order of stimulus presentation varied randomly across subjects.

### Data Analysis

The individual values of average heart rate and blood pressure (both diastolic and systolic) recorded during the various music conditions underwent three ANOVAs with two factors of variability: music style (atonal vs. tonal), and emotion (agitating, joyful, touching). Tukey’s *post hoc* test was used for comparisons across means.

## Results

**FIGURE 4:**
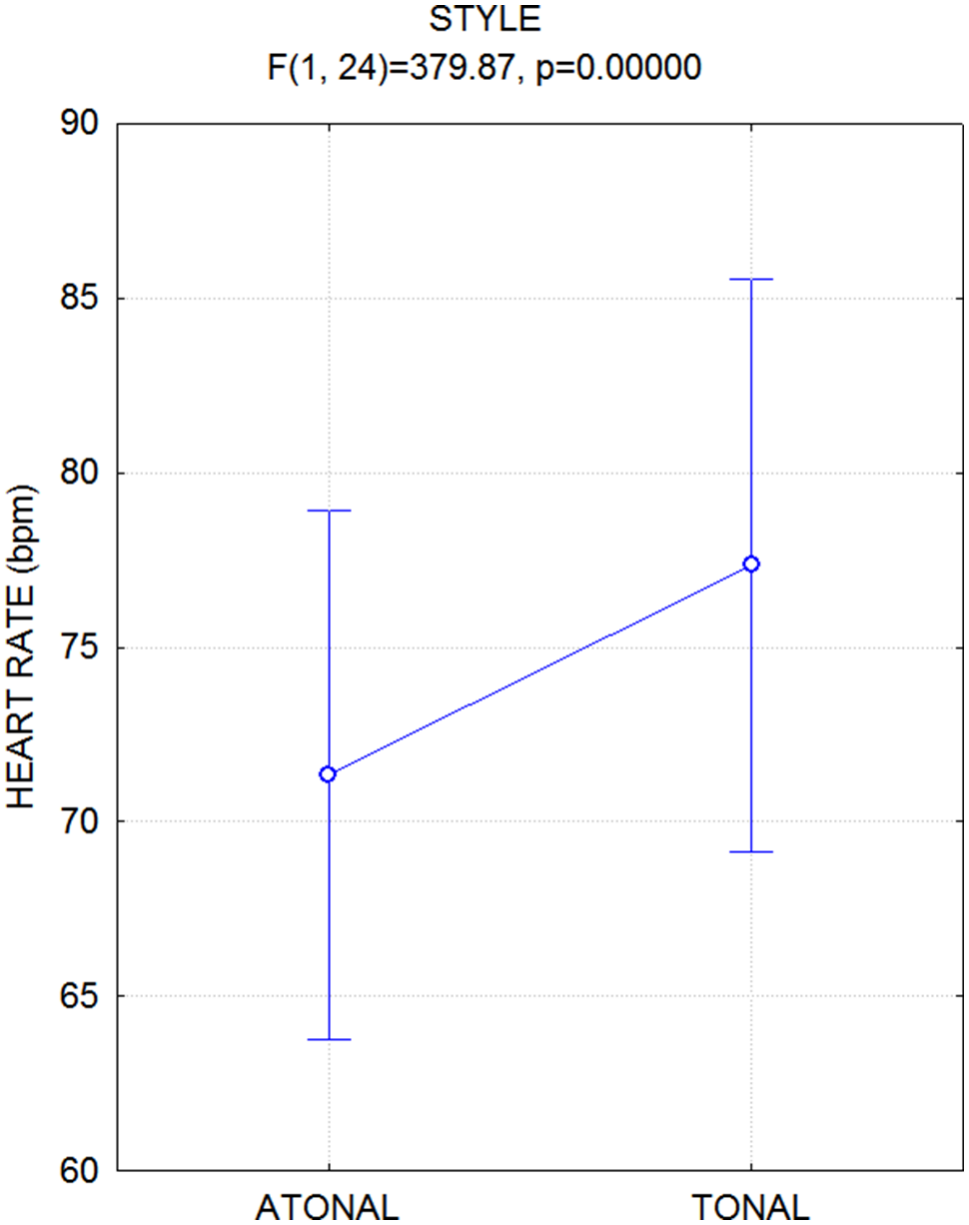
**Mean values of heart rate (BPM), along with standard errors, recorded while participants listened to atonal vs. tonal music pieces**.

**FIGURE 5:**
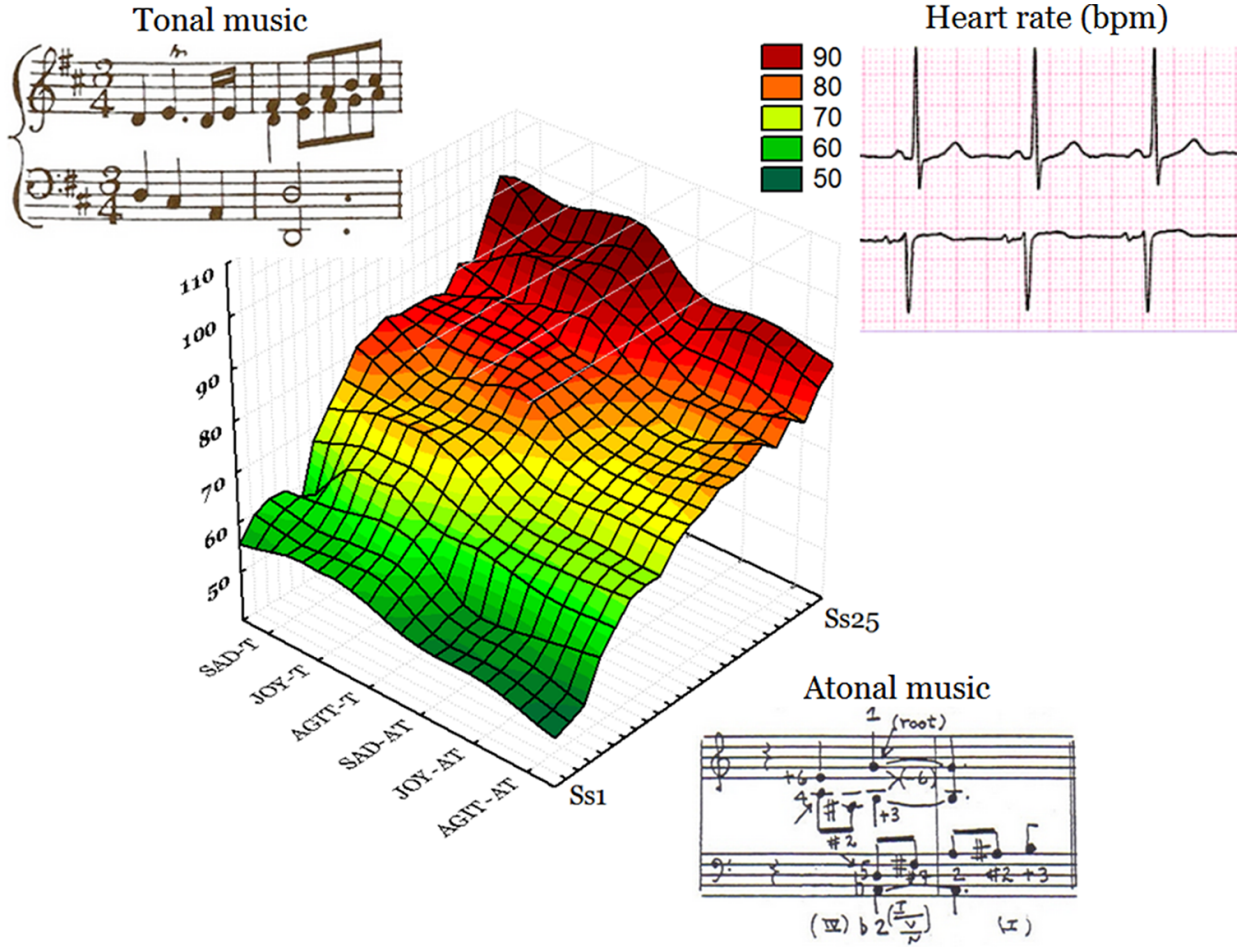
**Individual values of heart frequency recorded in healthy university students while listening to tonal or atonal musical pieces.** The latter condition was associated with a pronounced bradycardia (represented by a more extended green and yellow area). AGIT-AT = atonal agitating; JOY-AT = atonal joyful; SAD-AT = atonal touching; AGIT-T = tonal agitating; JOY-T = tonal joyful; SAD-T = tonal touching.

The ANOVA performed on heart rate values yielded the significance of music style [*F*(1,24) = 380; *p* < 0.000001], and heart frequency was lower while listening to atonal (71.3, *SD* = 2.18 bpm) over tonal music (77.4, *SD* = 2.18 bpm), as visible in **Figure 4**. **Figure 5** displays the individual values of heart frequency recorded in healthy university students while listening to tonal or atonal musical pieces. No effect of emotion was visible except for a tendency of touching music to increase heart rate, particularly atonal music, as indicated by the significant Emotion × Style interaction [*F*(2,48) = 6.39; *p* < 0.0035], and relative *post hoc* comparisons.

**FIGURE 6:**
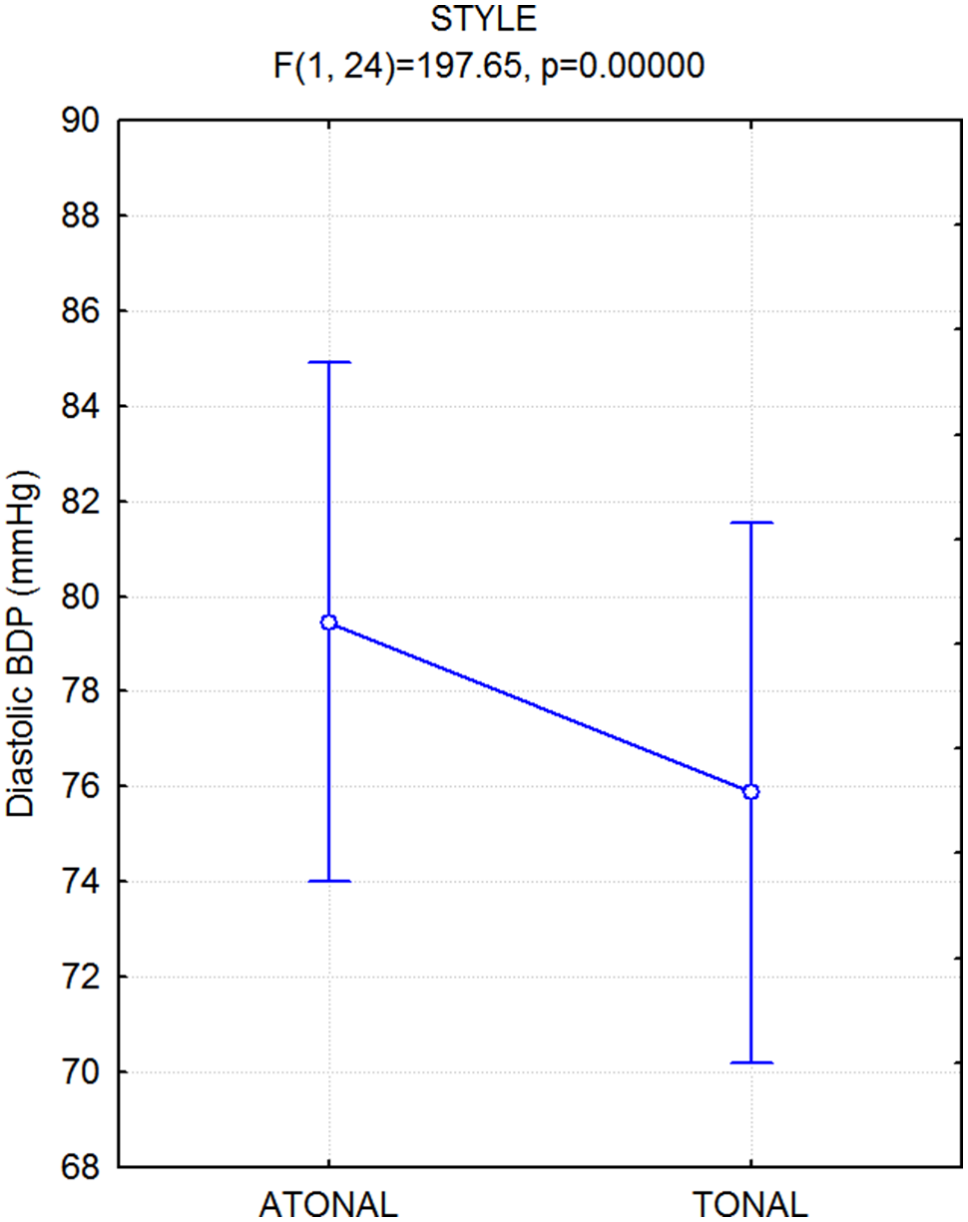
**Mean values of diastolic blood pressure (diaBDP), along with standard errors, recorded while participants listened to atonal vs. tonal music pieces**.

The ANOVA performed on diastolic blood pressure values yielded the significance of stimulus type [*F*(1,24) = 198; *p* < 0.000001], and sysBLP was higher overall while listening to all atonal vs. tonal pieces (see **Figure 6**). There was no significant effect of emotion.

**FIGURE 7:**
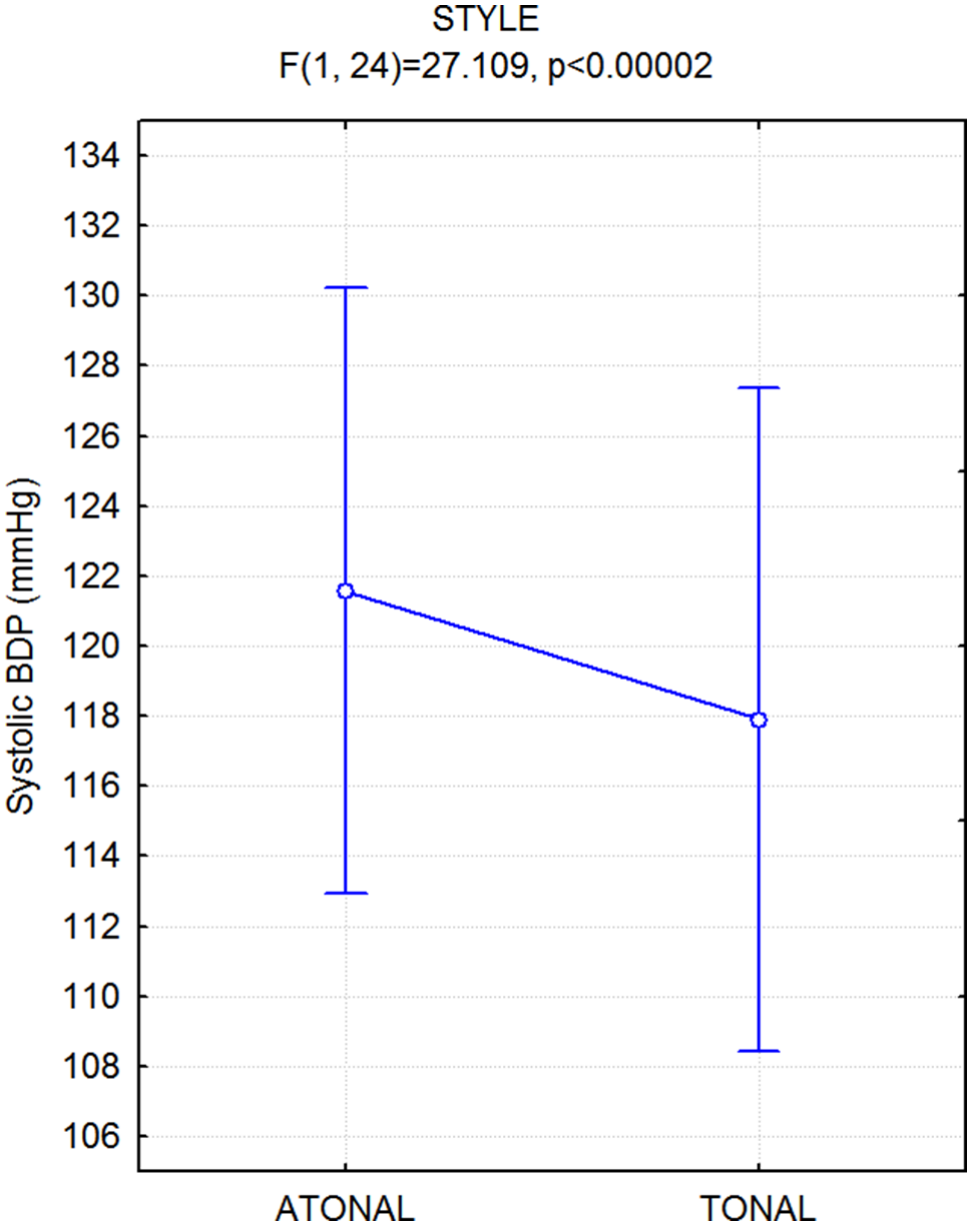
**Mean values of systolic blood pressure (sysBDP), along with standard errors, recorded while participants listened to atonal vs. tonal music pieces**.

The ANOVA performed on systolic blood pressure values yielded the significance of music style [*F*(1,24) = 27.11; *p* < 0.000025], and sysBLP was higher overall while listening to all atonal rather than tonal pieces (see **Figure 7**). There was no significant effect of emotion.

## Discussion

Listening to instrumental atonal music (independent of the piece’s emotional characteristics) was associated with reduced heart rate (bradycardia) and increased blood pressure (both diastolic and systolic) compared to the tonal condition. Bradycardic changes of heartrate are associated with *interest* and *attending* to a stimulus (Coles and Duncan-Johnson, 1977; Hernández and Watson, 1997). Furthermore, bradycardic changes and increased blood pressure are associated with anxiety, tension and lack of relaxation. It is therefore possible that listening to atonal music might induce a parasympathetic response whose peripheral effects are sensed by the listener to be psychological tension and agitation. Indeed, according the esthetic assessment made by professional conductors and composers, the atonal repertoire is by definition more agitating. This interpretation fits very well with the findings commonly reported of a deceleration of the cardiac activity during perception of scary, threatening or noxious sounds. This defensive response is called fear-induced bradycardia, and is observable also in newborns (Anderssen et al., 1993). However, the psychophysiological literature is conflicting on this matter (i.e., the effects of agitating music on autonomic responses). For example, Hilz et al. (2014) monitored systolic and diastolic blood pressure while participants either sat in silence or listened to “relaxing” vs. “aggressive” excerpts of classical music. The results showed that listening to relaxing classical music and listening to aggressive classical music both increased systolic blood pressure, whereas the autonomic activation was lower under conditions of silence; therefore, aggressive music did not specifically increase blood pressure. The musical pieces were all tonal in this study, which might explain the lack of *fear* bradycardia. Our hypothesis is that the autonomic modulation found here is specifically related to the fearful aspects of atonal music, which was lacking in the music stimulation usually provided in previous psychophysiological studies.

Overall, by integrating psycho-esthetic with psychophysiological data from the present study, it can be hypothesized that the atonal music perception increased arousal and alertness to a greater extent than tonal music to the ears of non-musicians, as influenced by their auditory complexity and threatening properties. These notions are well-known to composers of thriller and horror movie soundtracks who make large use of dissonance and other expedients to scare audiences and create a sense of unease or dread (e.g., unresolved dissonances, timbral experimentation, unexpected recontextualization of a consonant and familiar-sounding musical work, atonality, stinger chords, repetitious drones, sudden changes in intensity, unpredictability, clashing dissonances; Lerner, 2009; Heimerdinger, 2012). These auditory stimuli have threatening and alerting properties, and may activate the amygdala, increase blood pressure, trigger an autonomic fear-related response, increase attention and excitability, and provide a psychological sense of anxiety (Davis and Whalen, 2001). **Table 2** shows a musicological description of our atonal stimuli, suggesting their ability to create an intensely emotional, moving, threatening or wondrous feeling in the listener (regardless of their specific emotional label).

**Table 2:** Musicological characterization of atonal pieces used in the psychophysiological study. Aside from their being associated with a specific emotional state (i.e., joy, agitation and pathos, respectively) they shared some properties (described below), such as their ability to induce anxiety and psychological tension in listeners, along with their atonality.

Hindemith, Paul First Movement of I Kammermusik (Joyful)
Kammermusik (1922) reflects Hindemith’s conception, typical of the 1920s, about the idea of composing functional music (*Gebrauchsmusik*) based on the invention of simple figurations and on repetitive-type motor rhythmics. There is the reference to Bach’s counterpointistic writing, although mediated by the use of modern materials. The orchestral ensemble envisaged – flute, clarinet, bassoon, trumpet in Bb, percussion, piano, accordion, and string quintet – in fact refers to jazz- and cabaret-like sonorities typical of that period. In the score, Hindemith characterizes the 1st movement with the following wording: *Sehrschnell und wild*, translatable as “very fast and wild” to refer to the agitated, repetitive and rhythmic nature of the musical writing used. The melodic-harmonic material is based on the stratification of diatonic-modal, defective scalar formulas, on bichords of perfect fifths and, in certain points, on the overlapping of different triads; overall, these elements give the reiterated, incisive and well-marked writing of rhythmic-melodic structures a generally euphonic sonority. The polarized use of heights – fixed, depending on the sections, on certain areas of the frequency space – generates an apparent and ambiguous stability as it is constantly thwarted and destabilized by the play of displacements, contrasts, and rhythmic deformations. The opening passage (corresponding to the selected 1-min fragment) is punctuated by rapid and vigorously swirling repeated quatrains of sixteenths by two violins and viola, combined with the sextuplets of piano, all in fortissimo; simultaneously, flute, clarinet, accordion, and cello (on a high-pitched sound range) perform in unison the short main motive, always repeated in fortissimo, and rhythmically interspersed at certain points by the trumpet. A second part of the composition is timbrally characterized by the punchy sound of the xylophone. As a whole, this first movement is articulated by means of short rhythmic-melodic formulas that are precise and clear, and which alternate without continuity between them, with resumptions subjected to deformations, generating asymmetry effects. In certain moments, the writing takes on a vehemently percussive character, thus further emphasizing the incisive and energetic nature of the piece. All this gives to the listener the feeling of cinematic music in which the motor rhythmicity predominates over all other musical elements.

**Donatoni, Franco** **Duo Pour Bruno (Agitating)** Duo for Bruno is an orchestral piece composed by Franco Donatoni approximately in 1974–1975. It is a tribute to Bruno Maderna, who had passed away on November 13, 1973. The starting material, in fact, is derived from a popular Venetian song “The blonde in gondoletta,” widely used by Maderna in his 1972 “Venetian Journal” for tenor, magnetic tape and orchestra. This diatonic material, however, is distributed by Donatoni between instruments without ever being quoted verbatim, except in the early parts of the work with the very dilated values of the oboes. The orchestra used by Donatoni does not include bassoons and tuba, and lower strings are reduced, thus resulting in a timbric lightening of the overall sonority. The title alludes to the duo’s instrumentation and to the formal structure of the work. There are two percussionists arranged symmetrically, two solo violins, celesta and vibraphone, two pianos, two harps and woodwinds and brass are treated in copies; each instrumental family is also divided into low- and high-pitched sounds. From a formal point of view, Duo for Bruno is divisible into 10 “panels,” often seemingly unrelated to each other and processed by various manipulations of varying complexity regarding their underlying structures. Each “panel” is conceived to be a diptych divided into 13 bars, a central bar acting as a hinge and 13 other bars, and it is characterized by its instrumental combinations, the plot of its internal representations, and its rhythmic articulations. Overall, in the succession of the 10 “panels,” a dynamic growing is felt, culminating in the finale, with a persistent, repetitive and “ostinato” TUTTI, punctuated by the powerful shots of the two bass drums alternately. At the same time, in each panel, the central bar, acting as a hinge between the two main parts, takes on a more and more disturbing valence. In it, in fact, the two solo violins and tubular bells intervene constantly. Starting from the second “panel,” the two violins overflow in bars around the central bar. This process, creating agitation and internal instability, increases more and more on the succession of “panels” to completely dissolve the hinge function at the end of the piece. The 1-min fragment played in the psychophysiological experiment coincides with part of the ninth “panel” characterized by its dissonant and violent colors, for the powerful tones of the four trombones, and for the immediate dynamic contrasts and sudden overturning. In the hinge bar, we hear seven notes of the bells and the isolated violin sounds. The descending chromatic melodic contours evoke the idea of pain or lamentation in the listener, and shifts toward the lower registers for mimesis suggest feelings of distress or suffering and transience. A state of intense and irrepressible excitement predominates, in which the hinge bar is followed by chord blocks in trill and tremolo by strings, alternated with polyphony of winds. These features can therefore generate a feeling of intense agitation and distress, with furious moments alternated with plaintive states. It must be remembered, however, that for Donatoni, Duo for Bruno represented a work mainly marked by a strong creative vitality and energy.

**Pärt, Arvo** **Cantus in Memoriam of Benjamin Britten (Touching)**
The composition of the Cantus dates back to 1977 and it is a type of funeral elegy that Arvo Pärt dedicated to the memory of Benjamin Britten, who passed away a year before, as suggested by the title itself. In this composition, the presence of that style that Part himself would later called “tintinnabuli” is recognizable, with reference to the resonances of the bells. It is a compositional style characterized by an extreme simplification of the harmony and the musical material used. As explained by the composer himself, if you strike a bell several times, a peculiar harmony will follow, oscillating around a low-pitched frequency. The “tintinnabuli” technique precisely reproduces these fluctuations, translating them into sequences and superimpositions of melodic lines, creating layers of harmonics. For example, the three notes of a triad can be interpreted, according to Pärt, as the sounds of a bell. Another feature is the use of the presence of two voices, one of which serves as an accompaniment by repeating the notes of the tonal chord, whereas the other carries the main melody. This is what happens in the Cantus, where the strings (except for violas) are divided mainly into two groups, except for violas. The fragment used in the study was taken by the initial piece of the work (after the first 15 s). The Cantus’s incipit is characterized by isolated bell sounds that remain constant after the entry of strings, regularly performing groups of three shots in a gradual crescendo toward *fff* to progressively decrease until the last stroke in *pp*, which ends the piece. Stringed instruments enter one after the other, from the first violins up to double bass, each on a same melody based on a minor natural descendent scale but in augmentation of a 1, 2, 4, 8, 16 ratio; this means that, for example, the double basses play the main melody augmented sixteen times compared to the figurations of the first violin. It is a canonical proportional technique, as was in vogue for the Flemish and Renaissance polyphonists. The first violins in the first part begin on sharp A5 and then repeatedly drop according to a rhythm in six quarters, adding a note to the lowest pitch of the scale; on the other hand, the first violins in the second part move along the notes of A minor arpeggio. The same is repeated with values dilated in other instruments, thus creating a harmonic-melodic texture on the notes of the eolic diatonic scale, like a sort of downward spiral projected simultaneously on several levels. In conjunction with the growing of the strokes of the bell, the arches gradually culminate over a *fff*, which remains constant until the conclusion. All of this is completed by gravitating on a long conclusive chord in A minor at full volume, suddenly leaving room for the slight distant resonance, at the edge of silence, of the last very delicate bell tolling. The Cantus, therefore, is essentially a moving meditation on death, symbolized by the initial silence from which the toll of the funeral bell intermittently emerge (alluding to the passing of Britten), by repeated descending movements toward a deep register, which pervade the entire work in their essential simplicity. This is also symbolized by the sound atmosphere of the minor mode and by the final silence with which the piece ends at its climax. In Arvo Pärt, all of this takes on a spiritual meaning related to his intimate religious faith.

The present findings show that the various music-induced emotions have a small modulatory effect on the vegetative response of ANS, in agreement with other psychophysiological studies providing negative (e.g., Etzel et al., 2006; Radstaak et al., 2014; Krabs et al., 2015), or inconsistent results (Iwanaga et al., 2005; Bernardi et al., 2006; Sammler et al., 2007; Tan et al., 2015). This pattern of autonomic responses suggests a fundamental difference with the cerebral responses to music, known to be clearly affected by the piece’s emotional connotation (Sammler et al., 2007; Salimpoor et al., 2011; Trost et al., 2012; Stupacher et al., 2013).

However, other data from our lab (Proverbio et al., 2015) with a larger group indicate that listening to touching musical pieces slightly but significantly increases heart rate (regardless or tonality) compared to environmental sounds (e.g., rain and thunder), suggesting that music is able to induce affective feelings regardless of compositional style. Other investigations are needed to determine the neural correlate of brain activation during music listening, which will likely highlight major differences as a function of music’s affective connotation (joyful vs. agitating vs. touching).

One of the limits of the study is the possibility of a culturally mediated difference in the esthetic preference for a tonal or atonal repertoire between the judges (who characterized the pieces as joyful, touching, and agitating) and naïve participants who listened to the selected pieces. Indeed, esthetic interviews were distributed to professional conductors and composers, who might have developed a positive esthetic taste for atonal music as a result of their specific profession. These judges concluded that the tonal and atonal repertoire was equally full of pathos and overall touching. On the other hand, we missed the esthetic evaluation of pieces from university students, who showed a strong difference in their autonomic responses to the tonal and the atonal pieces, possibly reflecting increased alertness and attention levels, a sense of wonder (feeling allured and amazed), psychological tension, and possibly anxiety and fear of the latter. The other possible limit relies in the concurrent visual stimulation. Indeed participants watched a sequence of 300 faces (balanced for affective valence and perceived arousal across auditory blocks) as a secondary task. Therefore they did not specifically pay attention to the auditory stimulation. If, on one hand, one might fear a cross modal interaction between auditory and visual processing (Marin et al., 2012), on the other hand, the inattentive auditory condition makes even more relevant the strong modulation of autonomic responses (fear bradycardia) which was found during listening to atonal music.

## Conflict of Interest Statement

The authors declare that the research was conducted in the absence of any commercial or financial relationships that could be construed as a potential conflict of interest.

## Footnotes

1 The *Cantus in Memoriam of Benjamin Britten* is characterized by the descendant nature of the main melody (in A minor), structured in compound time (six quarters), and which continuously repeats extending over the course of its subsequent reinstatements. This obsessively declining directionality, so marked and persistent from the beginning to the end of the piece, calls to mind the combination of beauty and transience to which it clearly refers. A partial analogy with Largo from the second movement of Bach’s double concerto is precisely due to the same downward direction of the main melody, also in compound time (12 eighths) although it takes place on a F major rather than A minor scale. The structural similarity between Part and Bach is suggested by the imitative style of both compositions: Bach melody takes place through some sort of “airy,” relaxed and lyrical dialog between the two concertante solo violins that alternate and overlap by counterpointing between each other. Similarly, in Part, lines overlap by organizing themselves two by two, forming however a complex heterophonic texture of the whole, given that his writing brings already into play the “tintinnabuli” style based on the resonance of the bell. Conversely, a piece like *The Unanswered Question* by Charles Ives, though beginning with a suspended and rarefied sonority due to the sustained notes of the strings (which is apparently reminiscent of some of Pärt’s atmospheres) is actually designed in a radically different way. Ives, in fact, works with a heterogeneous and layered material due to the brass atonal inserts that contrast *ex abrupto* with the “timeless” strings’ immobility, while Part’s heterophonic accumulation tends to greater fusion rather than to a contraposition as with the American composer. Part’s references to the canonical Flemish tradition thus motivate the pairing of his Cantus with Bach, rather than Ives or *Mahler’s Fifth Symphony Adagietto*. The Adagietto, in F major, limited to the use of strings with harp, is actually a Lied or a romance written for Alma Schindler. This piece is traditionally structured in ternary form and reaches its peak just before the closing, from which the rondò-finale immediately follows. Just its character of romanza (song), mainly supported by the wide melody of the first violins, keeps away Mahler’s writing of adagietto from the strictly canonical setting of Part’s Cantus in Memoriam.
